# An Application of Throughput Request Satisfaction Method for Maximizing Concurrent Throughput in WLAN for IoT Application System †

**DOI:** 10.3390/s24072173

**Published:** 2024-03-28

**Authors:** Bin Wu, Nobuo Funabiki, Sujan Chandra Roy, Md. Mahbubur Rahman, Dezheng Kong, Shihao Fang

**Affiliations:** Graduate School of Natural Science and Technology, Okayama University, Okayama 700-8530, Japan; ppxf92cc@s.okayama-u.ac.jp (B.W.); p7ku89oh@s.okayama-u.ac.jp (S.C.R.); mahbubur@jkkniu.edu.bd (M.M.R.); dezhengkong111@outlook.com (D.K.); pkpb8c9q@s.okayama-u.ac.jp (S.F.)

**Keywords:** Raspberry Pi, WLAN, traffic shaping, access point, target throughput, throughput maximization, high-density IoT networks

## Abstract

With the wide applications of the *Internet of Things (IoT)* in *smart home* systems, *IEEE 802.11n Wireless Local Area Networks (WLANs)* have become a frequently chosen communication technology due to their adaptability and affordability. In a high-density network of devices such as the *smart home scenerio*, a host often meets interferences from other devices and unequal *Received Signal Strength (RSS)* from *Access Points (APs)*. This results in *throughput unfairness/insufficiency* problems between hosts communicating concurrently in WLAN. Previously, we have studied the *throughput request satisfaction method* to address this problem. It calculates the *target throughput* from measured single and concurrent throughputs of hosts and controls the actual throughput at this target one by applying *traffic shaping* at the AP. However, the insufficiency problem of maximizing the throughput is not solved due to interferences from other hosts. In this paper, we present an extension of the *throughput request satisfaction method* to maximize the throughput of a high-priority host under concurrent communications. It recalculates the target throughput to increase the actual throughput as much as possible while the other hosts satisfy the least throughput. For evaluations, we conduct experiments using the test-bed system with *Raspberry Pi* as the AP devices in several topologies in indoor environments. The results confirm the effectiveness of our proposal.

## 1. Introduction

Nowadays, *IEEE 802.11 wireless local area networks (WLANs)* have been widely deployed around the world. WLAN has become the most popular way to access Internet services [1,2] since it offers cost-efficient, easy deployments and management in addition to mobility [3]. A nearby *access-point (AP)* enables a WLAN user to wirelessly access the Internet. As the *Internet of Things (IoT)* continues to expand, an increasing number of devices are connecting to networks via *WiFi* technology, which has found widespread application across various sectors, notably within *smart home* scenarios [4]. Despite the emergence of newer standards, *IEEE 802.11n* retains a pivotal role in the smart home due to its well-established technology and comprehensive product ecosystem.

*IEEE 802.11n* adopts the *carrier sense multiple access with collision avoidance (CSMA/CA)* protocol. It is basically sensitive to the *channel occupancy time* [5], which will lower the channel utilization and impact the throughput. A host located closer to its AP can experience the higher *received signal strength (RSS)* compared to a host situated farther away. This discrepancy in RSS will lead to the *throughput unfairness* problem among multiple hosts that communicate concurrently with APs in WLAN. Especially, for smart home application scenarios, including a multi-device IoT system connected via WLAN, various factors such as disparate *Received Signal Strength (RSS)*, *channel overlaps*, and *channel leakages* among adjacent channels can significantly impact the throughput performance. They will lead to the *throughput insufficiency* problem among concurrently communicating hosts.

According to our previous study in the paper [6], in real-world indoor testing, we used the *TP-Link WN722N* adapter and maintained a good signal range with an approximate radius of about 10 m to 12 m (RSS greater than −70 dbm). By using the *throughput estimation method* mentioned in the paper [6], the average concurrent throughput for each device reached the least throughput requirement (1.5 Mbps) when the number of devices served was at eight. In some cases, the *throughput unfairness* due to the above factors can lead to situations where the throughputs of the required devices are less than the least requirements.

Previously, to address the *throughput unfairness/insufficiency* problem, we have studied the *throughput request satisfaction method*. This method consists of three steps: (1) it measures the single and concurrent throughputs of each host, (2) it derives the *target throughput* for each host from the measurement results, and (3) it controls the traffic flow of each host by employing the *traffic shaping* technique at the AP to meet the *target throughput*. However, as the number of hosts and APs increase in the high-density WLAN environment, this approach cannot solve the insufficiency problem of maximizing the throughput of the high-priority host, due to interferences from other hosts.

In this paper, we propose an extension of the *throughput request satisfaction method* by recalculating and reassigning the *target throughput* to increase the actual throughput of the target host and apply this method to the smart home IoT scenario. This method increases the *target throughput* through the following four steps:

(1) It measures the *single throughput* and the *concurrent throughput* for each host to calculate the constant *channel occupancy time*,
(2) Based on the *channel occupancy time*, it calculates the *target throughput* for each host,
(3) It increases the *target throughput* of the high-priority host, decreases that of each non-priority host, and measures the result as the final maximized actual throughput, and
(4) It controls the traffic flow of each host by employing the *traffic shaping* technique at the AP to meet the *target throughput*.

For evaluations of the proposal, we implemented it in the WLAN testbed system that uses up to four *Raspberry Pi* as APs and conducted extensive experiments with various network topologies in #2 Engineering Building and Graduate School Building in Okayama University, Japan. The experimental results show that, in the majority of scenarios, the achieved throughput of the target host consistently surpasses the initially predicted and favorable expected value. In situations observing substantial path fading and interference from other communication links, our method can enhance the maximal throughput of the target host when compared to the previous method.

The rest of this paper is organized as follows: Section 2 introduces literature reviews. Section 3 reviews technologies related to this study. Section 4 reviews our previous works of the *throughput request satisfaction method*. Section 5 proposes its extension for the throughput maximization. Section 6 evaluates the proposal through experiments. Section 7 analyzes and discusses the results of the experiments. And Section 8 concludes this paper with future works.

## 2. Literature Review

In this section, we explore related works in the literature. Numerous studies have investigated issues of TCP *unfairness/insufficiency* throughputs in WLAN and throughput-satisfying methods for throughput maximization.

In [7], Yu et al. explored the potential of radio-frequency energy harvesting to power nodes in wireless networks and provided insights on maximizing the throughput via transmit power allocation of energy transmitters. The authors formulate the *sum-throughput maximization* problem as a non-linear optimization problem, prove its convexity, and propose an efficient dual-gradient algorithm. The proposal concludes with simulation results and discussions.

Contrasting this with our proposal, we implement maximum throughput control for the specified selected host, where the effectiveness is verified through real test-bed experiments.

In [8], Khorov et al. proposed *SEBRA (SAND-Enabled Bitrate and Resource Allocation)* as a centralized algorithm designed for network-assisted video streaming in wireless networks. *SEBRA* operates on Wi-Fi *access points (APs)* to efficiently distribute the video bitrate among video clients and allocate the channel time. They focus on the optimization of the client’s allocated channel time based on the bitrate demand. They convert the problem into an *NP-hard* model and use *heuristic algorithms* to find the optimal solution to optimize the channel time and the resource allocation.

In contrast, we mainly focus on how to satisfy the different demands through the adjustment of the *channel occupancy time* required to realize the throughput. Our method involves the use of the *tc command* in the *Linux OS* to control the upstream and downstream traffics, which realizes the actual traffic controls.

In [9], Chen et al. proposed a *Target Wake Time Scheduling Scheme (TSS)* to maximize throughputs in IEEE 802.11ax WLANs. The *TSS* scheme considers *OFDMA-based* multiuser transmissions by minimizing resource contentions between doze stations (STAs) in each beacon slot. The authors investigated key aspects to avoid collisions and ensure the maximum throughput while saving power using a novel broadcast *Target Wake Time (TWT)* mechanism for the AP to make decisions on *Target Beacon Transmission Time (TBTT)* for each *TWT* requesting station to improve the overall throughput. Simulation results are presented to analyze the performance of the *TSS* scheme, and conclusions are given.

In contrast, our approach focuses on the *channel occupancy time* between the hosts and APs that are communicating, calculates the target throughput, and applies the traffic control to maximize the target throughput of the high-priority host. We verify the effectiveness of the approach in test-bed experiments.

In [10], Yagi et al. proposed two novel control methods using frame aggregations of *IEEE802.11n/ac* to achieve the maximum overall throughput and fairness between multiple WLANs in densely deployed environments. The proposed method adjusts the transmission frequency and the frame aggregation size within the range to minimize the error probability. The authors validated the effectiveness of the proposed approach using a network simulator *NS-3* [11].

In contrast, our method considers the channel occupancy time to be allocated with traffic shaping to improve the throughput without modifying the MAC protocol. We verify the effectiveness of the method in a real test-bed environment.

In [12], Kuran et al. proposed a scheduler that addresses an optimization problem for maximizing the overall throughput for both uplink and downlink *IEEE 802.11ax OFDMA* transmissions. The proposal develops a throughput-maximizing scheduler considering channel bandwidths, STA traffic queues, and STA MCS levels. To prevent certain STAs with poor channel conditions from experiencing starvations, the authors incorporate an aging mechanism. Using the *NS-3* simulator [11], they evaluate the performance of the proposal in scenarios involving UDP and TCP traffic.

In contrast, our proposal achieves the desired throughput among competing hosts, instead of the uplink/downlink fairness. Moreover, our approach is implemented on a real testbed system without the need for modifying the MAC protocol.

In [13], Wu et al. proposed a high throughput resource unit assignment scheme for OFDMA-based WLANs. The proposed scheme uses a uniform subcarrier allocation algorithm to assign subcarriers to users, and a dynamic power allocation method to optimize the transmission power level for each user. Specifically, the scheme allocates subcarriers to users based on channel qualities and adjusts transmission power levels to minimize interferences and maximize throughputs. The authors conducted simulations to evaluate the performance of the proposed scheme.

In contrast, our proposal achieves throughput maximization using the traffic control command for adjusting the channel occupancy time.

In [14], Tewari et al. investigated a method that jointly assigns frequency and controls associations among STAs to maximize the aggregate throughput in WLAN. This approach comprises two primary steps. Firstly, an iterative algorithm is employed to allocate the optimum frequency to each STA in the network by regulating associations. Secondly, a heuristic algorithm is applied to fine-tune the frequency allocation scheme and maximize the overall throughput of all STAs. The simulation results demonstrate that the approach substantially enhances network throughput.

In contrast, our proposal involves adjusting the channel occupancy time to meet the throughput requirement of the chosen host. We conduct real test-bed experiments to validate the efficacy of our approach.

In [15], Djuraev et al. presented a channel hopping-based interference resilience scheme for wireless local area networks that considers throughput and fairness. The proposed scheme uses a channel hopping-based technique to reduce the packet loss rate due to interferences, minimize the impact on the network performance, increase the throughput, and promote fairness.

In contrast, our proposal addresses the unfair throughput problem among hosts and ensures the required throughput. It is implemented on the AP using standard Linux commands.

In [16], Kassa et al. proposed a novel method to optimize the frame size of the wireless local area network (WLAN) in downlink *multi-user multiple-input multiple-output (MUMIMO)* channels. The authors use a machine learning approach based on the *artificial neural network (ANN)* to predict the optimal frame size for each user based on the *channel state information (CSI)*, to optimize the throughput for each host. The proposed method is evaluated through simulations where the results show that this ANN-based approach outperforms the other methods, achieving the higher throughput and lower delay.

In contrast, our proposal involves adjusting the channel occupancy time and does not need to modify the MAC protocol.

In [17], Park et al. introduced a rapid link rate adaptation algorithm for WLAN, emphasizing its design and implementation. This algorithm enables swift adjustment of the transmission rate in response to variations in channel conditions, such as signal strength or interference. It operates based on a continuous feedback mechanism that monitors the wireless channel’s quality and modifies the transmission rate accordingly. To ensure stability, the feedback mechanism incorporates a moving average filter to smoothen out fluctuations in channel quality. The effectiveness was validated through simulations and experimental tests, demonstrating its ability to promptly adapt to changing channel conditions while maintaining a high throughput.

In contrast, our proposal calculates the target throughput based on the channel occupancy time in response to actual throughput and achieves the throughput control by traffic shaping.

## 3. Related Technologies

In this section, we introduce related technologies to this study.

### 3.1. Traffic Shaping

*Traffic shaping* controls the network bandwidth by managing the scheduling, policing, shaping, and classification of traffic. In Linux, the *tc* command can be used for this purpose. It consists of queueing discipline (qdisc), classes, and filters [18].

The *classful HTB qdisc* has been employed in our study to control traffic at the specific rate given by date rate $d_{i}$. The HTB algorithm uses *token buckets* to allocate traffic to classes based on two parameters *ceil* and *rate*, specifying the allocated and maximum bandwidth, respectively. Both parameters are set to the same value in this study. This approach ensures the desired quality of service for different traffic classes.

### 3.2. PI Controller of Rate and Ceil Parameters

In the realm of traffic shaping, the rate parameter value $d_{i}$ governs the maximum bandwidth allocation for a host. However, it is important to note that it cannot ensure the attainment of the specified throughput. To achieve the desired throughput, the *PI (Proportional-Integral) feedback control* is employed. This control mechanism dynamically updates the value of $d_{i}$ using the following equation, enabling the adaptive adjustment for the optimal throughput alignment:

(1) $$\begin{array}{c} d_{i}\left(m\right)=d_{i}\left(m-1\right)+K_{P}\times \left(R_{i}\left(m-1\right)-R_{i}\left(m\right)\right)\\ +K_{I}\times \left(t_{i}-R_{i}\left(m\right)\right). \end{array}$$

where $R_{i}\left(m\right)$ represents the actual throughput result at time step *m*, and $K_{P}$ and $K_{I}$ represent the *P-control* and *I-control* gains, respectively. $K_{P}=0.3$ and $K_{I}=0.7$ are adopted in this paper).

### 3.3. Throughput Measurement Tool

*iperf* is employed to generate the traffic needed for throughput measurement and measure the throughput [19]. By utilizing *iperf* at both the APs and hosts, we can conduct comprehensive throughput measurements.

## 4. Review of Throughput Request Satisfaction Method

This section reviews our previous proposal of the *throughput request satisfaction method* and discusses its limitations.

### 4.1. Throughput Unfairness/Insufficiency Observation

**Figure 1:**
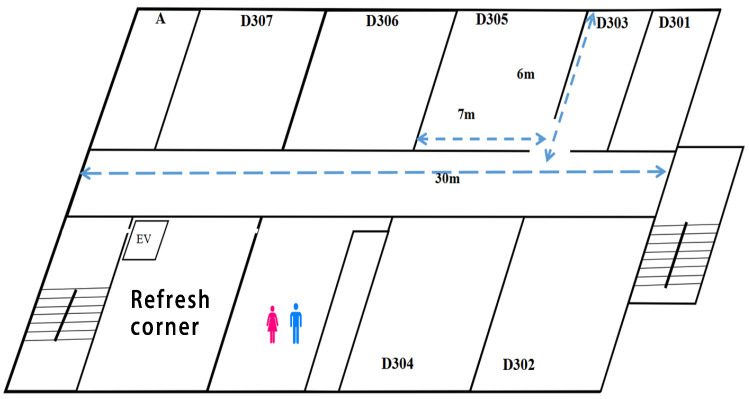
Indoor experiment field.

In WLAN, the *throughput unfairness/insufficiency* may appear among concurrently communicating hosts, due to disparate RSS as well as *channel overlaps* and *channel leakage* of adjacent channels. Previously, to investigate this issue, we conducted throughput measurements in indoor environments, specifically in the corridor of #2 Engineering Building at Okayama University. Figure 1 illustrates the experiment field. The measurements involve three cases:

(1) Two hosts are connected to the same AP using the same channel for the highest interference case.
(2) Two hosts are connected to different APs using the partially overlapped channels for the medium interference case.
(3) Two hosts are connected to different APs using the most distant channels for the least interference case.

**Figure 2:**
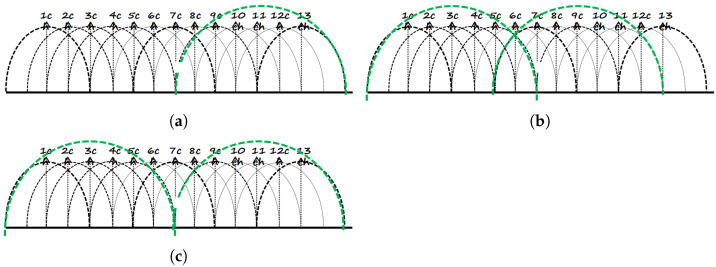
Channel assignment (**a**) Case 1; (**b**) Case 2; and (**c**) Case 3.

In any case, the hosts communicate with the *Raspberry Pi* AP through the *IEEE802.11n* 40 MHz bonded channels at 2.4 GHz. *Case 1* uses channels 9 + 13, *Case 2* uses channels 1 + 5 and channels 7 + 11, and *Case 3* uses channels 1 + 5 and channels 9 + 13. Figure 2 illustrates the channel assignment.

**Figure 3:**
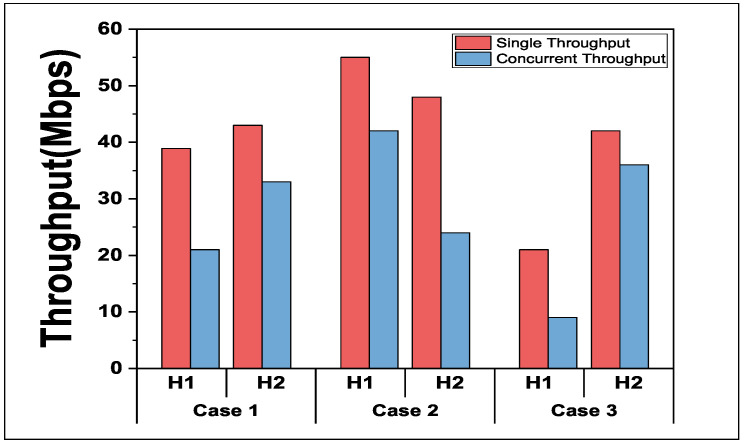
Throughput unfairness/insufficiency observations between two hosts.

Figure 3 shows the *single throughput* measurement results and the *concurrent throughput* ones for each host. The *single throughput* is measured when each host is communicating alone. The *concurrent throughput* is measured when all the hosts are simultaneously communicating with the APs. $H_{1}$ and $H_{2}$ have different RSS from the APs due to different distances and/or obstacles affecting them. *Case 1* involves the hosts sharing the same channel. *Case 2* involves the hosts using partially overlapped channels. *Case 3* involves the hosts using the most distant channels. The measurement results for each case indicate that a host receives an unfair throughput at concurrent communications due to different RSS, leading to potential insufficiencies for the hosts.

### 4.2. Throughput Request Satisfaction Method

To address the *throughput unfairness/insufficiency* problem, we have proposed the *throughput request satisfaction method* for concurrently communicating multiple hosts in WLAN [20,21]. Here, we briefly review it.

First, the two types of real throughput results, namely, the *single throughput* and the *concurrent throughput*, are measured for each host. The *single throughput* is measured when only the corresponding host communicates with the AP. It basically represents the *maximum throughput* of the host under no interference from other hosts in the WLAN. The *concurrent throughput* is measured when all the hosts simultaneously communicate with their associated APs in the WLAN. It represents the real throughput of the host under the interferences from the other hosts in the WLAN.

Next, the *channel occupancy time* of each host is calculated from the two types of throughput measurement results. It represents the time occupied by each host in the data transmission cycle where all the hosts transmit data by pulse periodically. Therefore, the sum of the *channel occupancy time* for all the hosts composes the cycle length that can be constant under concurrent communications of the hosts in the WLAN.

Third, the *target throughput* for each host is calculated based on the assumption that the sum of the *channel occupancy time*, or the cycle length, is constant when the *concurrent throughput* is replaced by the *target throughput*. The equation for calculating the *target throughput* is derived for each scenario of the throughput request.

Finally, the *traffic shaping* is applied at the AP to control the traffic of each host to achieve the *target throughput*. The *traffic shaping* is accomplished by adjusting the *data rate $d_{i}$* in the *tc command*.

In summary, the *throughput request satisfaction method* will apply the following steps:

(1) measure the *single throughput* of each host by communicating the host only,
(2) measure the *concurrent throughput* of each host by communicating all the hosts in the WLAN simultaneously,
(3) derive the *channel occupancy time* from the single and concurrent throughputs,
(4) calculate the *target throughput* for each host from the equation, and
(5) apply the *traffic shaping* to control the traffics of a host to achieve the *target throughput*.

### 4.3. Channel Occupancy Time Equation

To find the proper *target throughput* for each host, the *channel occupancy time* is derived from the measured *single throughput* and *concurrent throughput*. The *channel occupancy time* for the *i*-th host $H_{i}$ can be estimated by $\frac{C_{i}}{S_{i}}$. When all the hosts are communicating concurrently, the *channel occupancy time* of each host can be $\frac{C_{2}}{S_{2}}$, ..., $\frac{C_{n}}{S_{n}}$, and the sum of them will be a constant for the data transmission cycle. Then, if $C_{i}$ is replaced by $t_{i}$, the sum will still be constant. Therefore, using the symbols in Table 1, the following equation is derived:

(2) $$\begin{array}{c} \frac{C_{1}}{S_{1}}+\frac{C_{2}}{S_{2}}+\cdots +\frac{C_{n}}{S_{n}}=\frac{t_{1}}{S_{1}}+\frac{t_{2}}{S_{2}}+\cdots +\frac{t_{n}}{S_{n}}. \end{array}$$

### 4.4. Target Throughput Setting for High-Priority Host

In the previous study, a *high-priority* host is considered among the hosts such that this host requests the highest possible throughput, which is given by the single throughput of the host. In this scenario, under concurrent communications, the selected high-priority host $H_{k}$ should enjoy the single throughput result $S_{k}$ while the others should satisfy the equal minimum throughput result for fairness. Therefore, the following equations are derived for this scenario:

$$t_{k}=S_{k},$$

(3) $$t_{1}=t_{2}=\ldots =t_{n}\;\left(except\;for\;t_{k}\right)=\frac{1}{\sum_{\begin{array}{c} i=1 \end{array}}^{n}{\;}_{\begin{array}{c} i\neq k \end{array}}\frac{1}{S_{i}}}\left(\sum_{\begin{array}{c} i=1 \end{array}}^{n}\frac{C_{i}}{S_{i}}-\frac{t_{k}}{S_{k}}\right).$$

where $t_{k}$ is the target throughput for the high-priority host $H_{k}$.

### 4.5. Limitations

Unfortunately, our experiments revealed that the previous *throughput request satisfaction method* cannot achieve the request of the high-priority host when the number of hosts is large in the WLAN due to the high interferences. In the application scenario of the *smart home* IoT system, it is necessary to properly increase the *target throughput* of the high transmission demand devices such as PCs and smartphones, etc., while satisfying the minimum throughput constraint of the remaining hosts. Meanwhile, setting the *target throughput* of the high-priority host equal to its *single throughput* does not satisfy the lowest requirements of all hosts in some cases. In the following sections, to address the limitations, we propose an extension of the *throughput request satisfaction method* to satisfy the throughput request of the high-priority host as best as possible.

## 5. Extension for Throughput Request Satisfaction Method

In this section, we present the extension of the previous *throughput request satisfaction method* to maximize the concurrent throughput.

### 5.1. Idea of Extension

In this proposal, we set the specified host as the *high priority* device, to emulate the devices with high transmission requirements in the *smart home* IoT system.

To address the limitations of the maximum of the actual throughput in the previous approach, we propose the extension of implementing the two main steps to enhance the maximum throughput attainable:

(1) First, we set the least required throughput for the non-priority hosts as their *target throughput*.
(2) Second, we calculate the *increased target throughput* for the high-priority host.

Compared with the previous method, our proposal uses the *increased target throughput* for the high-priority host, instead of assigning its *single throughput*. In this way, it can provide all the hosts with the least required throughputs and give the high-priority host a much higher maximized actual throughput than before.

### 5.2. Extended Method Procedure

The procedure is outlined in the following steps:

(1) measure the *single throughput* for each host when only one host communicates with its associated AP,
(2) measure the *concurrent throughput* of all hosts when they communicate concurrently with their associated APs,
(3) calculate the *channel occupancy time* using the measured data,
(4) set the *target throughput* $t_{i}$ of the non-priority hosts to be equal to their least required throughputs,
(5) calculate the *increased target throughput* $T_{i}$ for the high-priority host based on the *channel occupancy time*,
(6) assign the *target throughput* $t_{i}$ and *increased target throughput* $T_{i}$ to the initial *rate* parameter $d_{i}$ for each host,
(7) apply *traffic shaping* while adjusting $d_{i}$ by the *PI control*, and
(8) measure the actual throughputs of all hosts.

Steps (1) to (3) align seamlessly with the previously proposed method, employing both single and concurrent throughput measurements to delineate their correlation with the *channel occupancy time*.

Step (4) allows all hosts to satisfy their lowest requirements. Step (5) calculates how much target throughput should be increased for the high-priority host. Compared with the previous method, this step involves the direct prioritization of the high-priority host, which should exhibit maximal throughput, and the computation of the *channel occupancy time* for the remaining hosts within the present channel communication condition.

Steps (6) and (7) involve the application of the *traffic shaping* technique, which adds a throughput filter to each host for effective control of throughput. These two steps also involve updating $d_{i}$ to ensure that the actual results closely align with the *target throughput* we set.

### 5.3. Increased Target Throughput Calculation

In this extension of the throughput maximum request method, considering the throughput reduction caused by the *Carrier Sense* and *Back-off* mechanism of *CSMA/CA* protocol, it is necessary to increase the target throughput under high traffic scenarios, to address the limitation of the previous methods mentioned in Section 4.5.

In our method procedure, the *target throughput* of the high-priority host will be increased and recalculated while all other hosts enjoy the lower throughput assignments. To facilitate the validation of the method, we set the least throughput of these non-priority hosts to 1.5 Mbps in this paper. That is, for example $H_{1}$ as the high-priority host, the *target throughput* of other hosts $t_{2}=t_{3}=\cdots =t_{n}=1.5$ Mbps, and the *increased target throughput* of high-priority host $H_{1}$ should be $T_{1}$. The equations are shown as follows:

(4) $$\frac{C_{1}}{S_{1}}+\frac{C_{2}}{S_{2}}+\cdots +\frac{C_{n}}{S_{n}}=\frac{t_{1}}{S_{1}}+\frac{t_{2}}{S_{2}}+\cdots +\frac{t_{n}}{S_{n}},$$

(5) $$t_{2}=t_{3}=\ldots =t_{n}=1.5\;\mathrm{Mbps},$$

(6) $$T_{1}=S_{1}\times \left(\sum_{i=1}^{n}\frac{C_{i}}{S_{i}}-\sum_{i=2}^{n}\frac{t_{i}}{S_{i}}\right).$$

## 6. Evaluation for Extension of Maximizing Throughput Proposal

In this section, we evaluate the extension method through experiments using the test-bed system.

### 6.1. Experimental Setup

**Figure 4:**
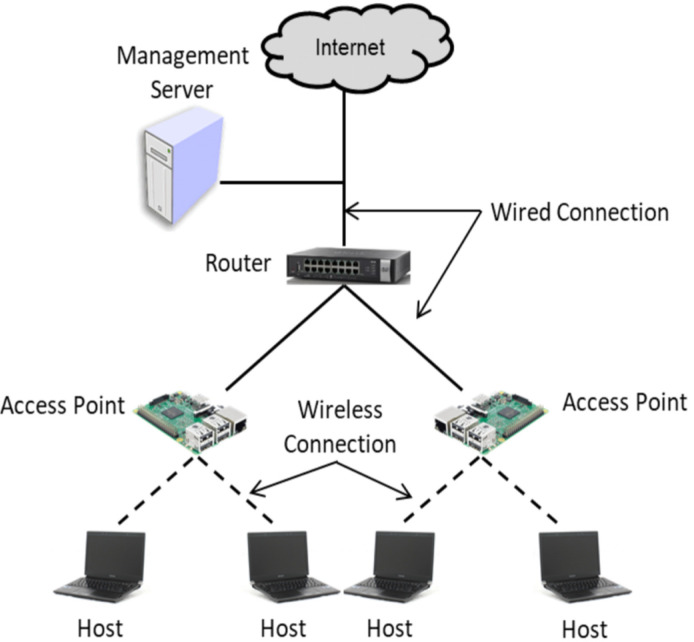
Topology of test-bed system.

In our experiments, we used two to four APs, where each AP is connected to one or two hosts, with the topology shown in Figure 4, to fully validate the effectiveness of the method for improving the maximum throughput. The *Raspberry Pi 3B*, equipped with the *TP-Link TL-WN722N* wireless NIC adapter [22], is utilized as the *Access Point (AP)*. *Linux-based* laptop computers are employed for the management server and the hosts. Table 2 shows the specifications of software and hardware in this test-bed system.

To enhance the accuracy of the measured throughput results, which exhibited frequent fluctuations, we employed a *repetition-based approach*. Each throughput measurement was conducted 10 times, and the average value obtained from these repetitions was utilized in evaluations. The duration of each measurement session was one minute, resulting in a total measurement time of 10 min per throughput.

### 6.2. Experiment Fields

**Figure 5:**
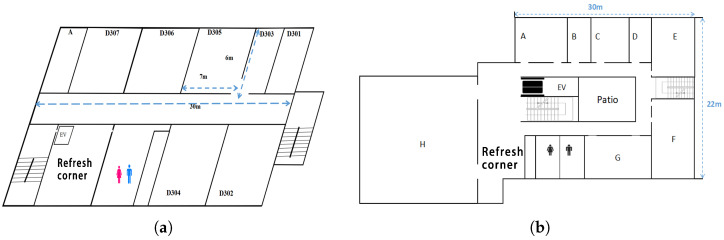
Experiment fields: (**a**) Engineering Building #2; (**b**) Graduate School of Natural Sciences Building.

The experiments were conducted in #2 Engineering Building and Graduate School Building at Okayama University, Japan, as shown in Figure 5.

### 6.3. Experiment Cases and Devices Locations

To assess the effectiveness of the proposed method across various channel occupancy scenarios, our experiments are categorized into three scenarios with two APs, three APs, and four APs, representing three application fields of *small space*, *medium space*, and *large space*. This setup ensures the effective coverage of the predetermined scenarios when the signal strengths are favorable. Meanwhile, as the number of APs increases, the factors affecting the throughput drop such as *channel leakage*, *RSS reduction*, and *channel overlap* will change. This arrangement allows the better verification of the method’s generalizability when applied in different situations.

Furthermore, each scenario was tested with one and two hosts connected to each AP, respectively. When only one host is connected to the AP, it is possible to verify the effectiveness of the method to solve the *unfairness/insufficient* throughput problem due to the difference in RSS, and under the interference of *channel leakage* as well as *partially channel overlapping*. The *full channel overlapping* interference is enhanced when two hosts are connected to the AP, and it is possible to verify the usefulness of the proposal for adjusting the throughput.

In all the scenarios, to achieve the objective of maximizing the throughput for high-speed communication needs, we employed the *iperf 2.0.5* tool to emulate the high-performance TCP connections in the created network environment. The configuration utilized the *TCP traffic* with a *window size* of 477 KB and a *buffer size* of 8 KB, testing the bandwidth of communication links for *smart home* devices throughout the house. This setup is instrumental in assessing the network performance under conditions of the high throughput and the low latency. It is particularly relevant for devices requiring high-speed connections, such as personal computers and network-enabled televisions, as the bandwidth outcomes from these measurements provide a clearer indication of whether the throughput demands of these devices are fulfilled. Although tailored for high-performance transmissions, this configuration adequately supports the basic communication needs of devices with small packet requirements, like smart light bulbs. At the same time, the distance of the host from the AP is set to different sizes to simulate the condition in real applications.

The APs and hosts locations are arranged as shown in the following Table 3, Table 4 and Table 5.

For all 16 cases, we summarize their connection assignments, application scenarios, and main reasons for the throughput drop being interfered with in the following Table 6, in order to have a better understanding.

## 7. Throughput Results and Discussions

In this section, we show the effectiveness of the extended method by discussing the results of the sixteen cases mentioned above. Additionally, by comparing with the previous methods, we display the effectiveness of addressing the limitations of ensuring the least required throughput of the non-priority hosts and the increased maximum actual throughput for the high-priority host.

### 7.1. Throughput Results of 16 Cases

**Figure 6:**
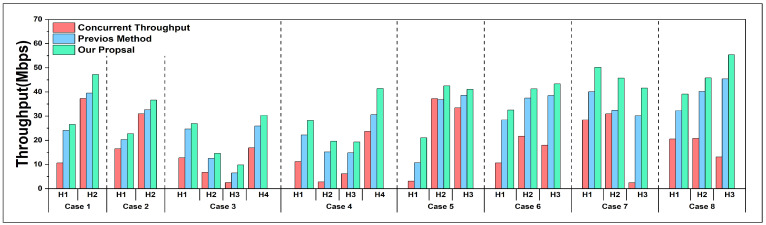
Results of *Case 1* to *Case 8*.

**Figure 7:**
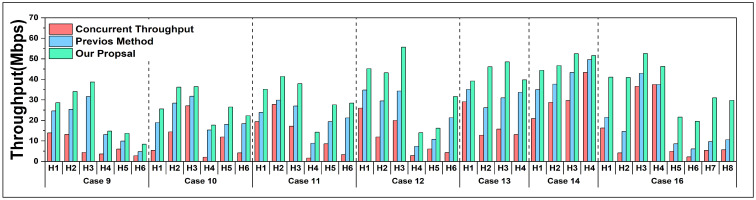
Results of *Case 9* to *Case 16*, except for special *Case 15*.

Figure 6 and Figure 7 present the experimental data for 16 cases except for *Case 15*. Every host was selected separately as the high-priority host for the throughput maximization. At the same time, other non-priority hosts were given with their least required throughput in each experiment. The columns in the figures separately represent the *concurrent throughput*, the results using the previous method, and the results after applying the proposal.

Particularly, in *Case 15*, the *target throughput* assigned for the non-priority host cannot satisfy the lowest requirement using the previous method. We will discuss and compare the result of *Case 15* with our proposal, in the following subsection.

### 7.2. Discussions

From the result data, it can be noticed that the throughputs have significant improvements by using our proposal.

In order to maximize the throughput of the high-priority host while communicating simultaneously, the previous method can be effective but still has space for improvement. After recalculating the increased target throughput for the high-priority host, we can observe that our proposed method can further maximize the actual throughput for the high-priority host, comparing the results obtained with the previous method.

**Figure 8:**
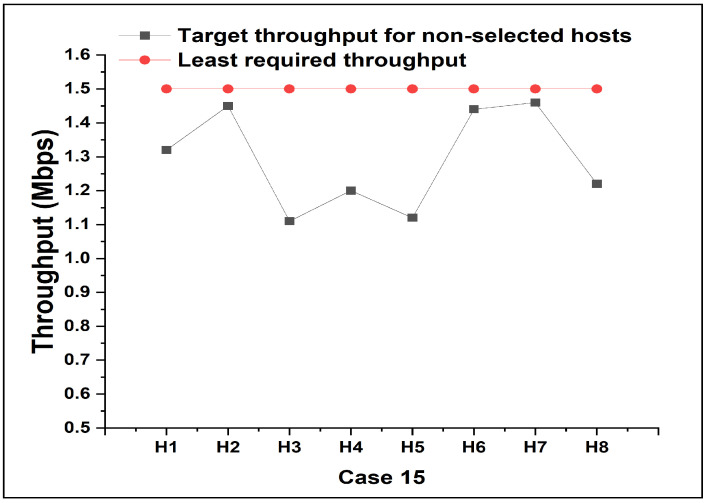
Target throughput results by previous method in *Case 15*.

**Figure 9:**
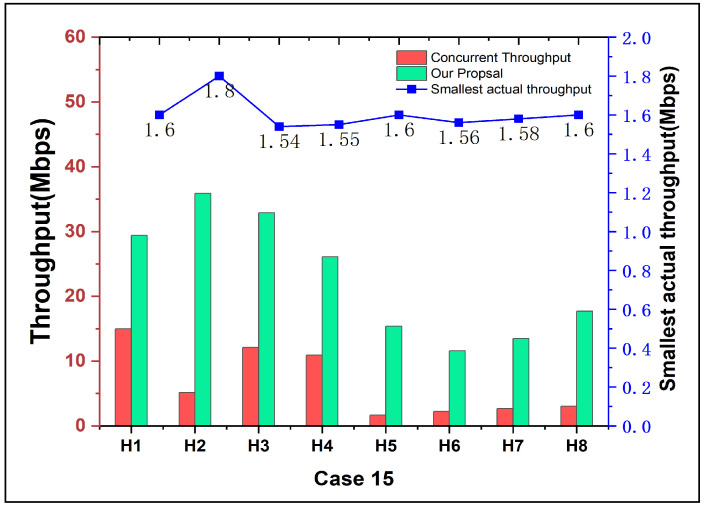
Actual throughput results by proposal in *Case 15*.

For *Case 15*, as depicted in Figure 8, the previous method gave lower target throughputs than the lowest requirement for the non-priority hosts, which gave the higher throughput to the high-priority host. The proposed method addresses this limitation by giving the least required throughput to all the hosts, as shown by the folded line in Figure 9. It shows that the smallest throughput during each experiment never falls below the given requirement (1.5 Mbps in this paper). The results in Figure 9 demonstrate the effectiveness of our approach.

## 8. Conclusions

This paper presents the extension of the *throughput request satisfaction method* to maximize the *concurrent throughput* of a high-priority host in the application of *smart home* IoT system. It increases the *target throughput* to improve the actual throughput for the high-priority host while providing the least throughput to the other hosts. For evaluations of the proposal, we conducted extensive experiments using the real test-bed system with *Raspberry Pi APs* and *Linux* hosts, with sixteen cases in two experiment fields at Okayama University. The results confirmed the effectiveness of our proposal. In future works, we will enhance this method by considering the *transmission power control* and evaluate the method with 5 GHz in various network scenarios.

## Figures and Tables

**Table 1:** List of symbols.

Notations	Definition
$H_{i}$	*i*th host for i=1,2,…,n, where *n* is the number of host
$S_{i}$	measured *single throughput* of $H_{i}$
$C_{i}$	measured *concurrent throughput* of $H_{i}$
$t_{i}$	*target throughput* of $H_{i}$

**Table 2:** Hardware and software specifications.

Access Point	
Model	Raspberry Pi 3B
CPU	BCM2837 1.2 GHz, Broadcom
RAM	LPDDR2 900 MHz 1 GB
Operating System	Linux Raspbian
Software	*hostapd*, *iperf 2.0.5*
External NIC	TP-Link WN722N
**Server PC**	
Model	Fujitsu Lifebook S761/C
CPU	Intel Core i5-2520M @2.5 GHz
RAM	4 GB DDR3 1333 MHz
Operating System	Linux Ubuntu 14.04 LTS (kernel 3.13.0-57)
Software	*iperf 2.0.5*
**Host PC**	
Model	1. Toshiba Dynabook R731/B 2. Toshiba Dynabook R734/K
CPU	1. Intel Core i5-2520M @2.5 GHz 2. Intel Core i5-4300M @2.5 GHz
RAM	4 GB DDR3 1333 MHz
Operating System	Linux Ubuntu 14.04 LTS (kernel 3.13.0-57)
Software	*iperf 2.0.5*

**Table 3:** Location of APs and hosts: two AP scenarios.

Case	Exp. Field	Location					
		${\mathrm{AP}}_{1}$	$H_{1}$	$H_{3}$	${\mathrm{AP}}_{2}$	$H_{2}$	$H_{4}$
*1*	EB2 *	Outside D307	D307	-	D307	D307	-
*2*	EB2	D307	Outside D307	-	D307	D307	-
*3*	EB2	D307	D307	Outside D306	D307	D307	Outside D307
*4*	EB2	D306	D306	Outside D306	D307	D307	Outside D307

* EB2 represents the experimental field #2 Engineering Building.

**Table 4:** Location of APs and hosts: three AP scenarios.

Case	Exp. Field	Location								
		${\mathrm{AP}}_{1}$	$H_{1}$	$H_{4}$	${\mathrm{AP}}_{2}$	$H_{2}$	$H_{5}$	${\mathrm{AP}}_{3}$	$H_{3}$	$H_{6}$
*5*	EB2 ^1^	Outside D308	Refresh corner	-	D307	D307	-	Outside D305	Outside D305	-
*6*	EB2	D307	D307	-	D306	D306	-	Refresh corner	Refresh corner	-
*7*	Grad. ^2^	Room H	Room H	-	Room H	Room H	-	Room H	Room H	-
*8*	Grad.	Room A	Room A	-	Room B	Room B	-	Room C	Room C	-
*9*	EB2	D307	D307	Outside D307	D306	D306	Outside D306	Refresh corner	Refresh corner	D306 and D307 corridor
*10*	EB2	Outside D308	Refresh corner	Refresh corner	D307	D307	D307	Outside D305	Outside D305	Outside D305
*11*	Grad.	Room H	Room H	Before stairs	Outside Room D	Outside Room D	Outside Room B	Before stairs	Before stairs	Refresh corner
*12*	Grad.	Room H	Room H	Outside Room H	Room A	Room A	Outside Room A	Room C	Room C	Outside Room C

^1^ EB2 represents the experimental field #2 Engineering Building; ^2^ Grad. represents the experimental field Graduate School of Natural Sciences Building.

**Table 5:** Location of APs and hosts: four AP scenarios.

Case	Exp. Field	Location					
		${\mathrm{AP}}_{1}$	$H_{1}$	$H_{5}$	${\mathrm{AP}}_{2}$	$H_{2}$	$H_{6}$
*13*	Grad. *	Room H	Room H	-	Outside Room C	Room C	-
*14*	Grad.	Room H	Room H	-	Room C	Room C	-
*15*	Grad.	Room H	Room H	Outside Room H	Outside Room C	Room C	Outside Room E
*16*	Grad.	Room H	Room H	Before stairs	Room C	Room C	Outside Room B
**Case**	**Exp.** **Field**	**Location**					
		${\mathrm{AP}}_{3}$	$H_{3}$	$H_{7}$	${\mathrm{AP}}_{4}$	$H_{4}$	$H_{8}$
*13*	Grad.	Refresh corner	Refresh corner	-	Outside Room A	Room A	-
*14*	Grad.	Refresh corner	Refresh corner	-	Room A	Room A	-
*15*	Grad.	Refresh corner	Refresh corner	Before stairs	Outside Room A	Room A	Outside Room B
*16*	Grad.	Room G	Room G	Before Toilet	Room A	Room A	Outside Room A

* Grad. represents the experimental field Graduate School of Natural Sciences Building.

**Table 6:** The application and connection assignments list for all 16 Cases.

Cases	Connection Assignment	Interference Factor	Application Scenarios
1 and 2	$AP_{1}$–$H_{1}$ $AP_{2}$–$H_{2}$	Channel Leakage	Small space with walls
3 and 4	$AP_{1}$–$H_{1},H_{2}$ $AP_{2}$–$H_{3},H_{4}$	Channel Leakage Full CO *	Small space with walls
5, 6, and 7	$AP_{1}$–$H_{1}$ $AP_{2}$–$H_{2}$ $AP_{3}$–$H_{3}$	Channel Leakage Partially CO	Medium space with walls
8	$AP_{1}$–$H_{1}$ $AP_{2}$–$H_{2}$ $AP_{3}$–$H_{3}$	Channel Leakage Partially CO	Medium space without walls
9 and 10	$AP_{1}$–$H_{1},H_{2}$ $AP_{2}$–$H_{3},H_{4}$ $AP_{3}$–$H_{5},H_{6}$	Channel Leakage Partially CO Full CO	Medium space with walls
11 and 12	$AP_{1}$–$H_{1},H_{2}$ $AP_{2}$–$H_{3},H_{4}$ $AP_{3}$–$H_{5},H_{6}$	Channel Leakage Partially CO Full CO	Large space with walls
13 and 14	$AP_{1}$–$H_{1}$ $AP_{2}$–$H_{2}$ $AP_{3}$–$H_{3}$ $AP_{4}$–$H_{4}$	Channel Leakage Partially CO	Large space with walls
15 and 16	$AP_{1}$–$H_{1},H_{2}$ $AP_{2}$–$H_{3},H_{4}$ $AP_{3}$–$H_{5},H_{6}$ $AP_{4}$–$H_{7},H_{8}$	Channel Leakage Partially CO Full CO	Large space with walls

* CO represents the *Channel Overlaps*.

## Data Availability

Data are contained within the article.
